# Multisensory vs. unisensory learning: how they shape effective connectivity networks subserving unimodal and multimodal integration

**DOI:** 10.3389/fnins.2025.1641862

**Published:** 2025-09-19

**Authors:** Ioanna Porfyri, Evangelos Paraskevopoulos, Alexandra Anagnostopoulou, Charis Styliadis, Panagiotis D. Bamidis

**Affiliations:** ^1^Lab of Medical Physics and Digital Innovation, School of Medicine, Faculty of Health Sciences, Aristotle University of Thessaloniki, Thessaloniki, Greece; ^2^Department of Psychology, University of Cyprus, Nicosia, Cyprus

**Keywords:** multisensory training, music training, EEG, neuroplasticity, effective connectivity

## Abstract

The brain synthesizes meaningful interpretations out of the surrounding environment, by integrating sensory input collected by multiple senses. Learning based on contextual multisensory stimulation is considered superior to unisensory. Multisensory methods implemented in rehabilitation and educational studies have demonstrated remarkable neuroplastic changes within cortical networks. However, the exact mechanisms underlying the ensuing neuroplasticity continue to elude comprehension. The present work intends to address this gap at the large-scale level by modeling the experience-induced alterations of multisensory and unisensory training in the effective cortical networks that subserve visual, auditory, and audiovisual information processing. Pre- and post-training EEG analysis demonstrated that the cross-modal training alters significantly the effective connectivity networks in all three modalities, whilst the unisensory methodological approach exerts impact solely on a unisensory (auditory) system. The regions that exhibit most of the alterations are identified within the left medial frontal gyrus (MFG), the left inferior frontal sulcus (IFS), as well as the left insula, areas with renowned multisensory properties. The reconfiguration of the connections following the multisensory training and during the visual and auditory integrative processes concerns mainly higher-order cortical areas, suggesting a top-down process affecting unisensory perception. The results of our study not only strengthen the theory of the superiority of multisensory training compared to unisensory but also indicate that the influence of multimodal training on the unisensory systems succeeds through feedback connections from higher-order association areas, highlighting the complexity of neurophysiological pathways of human perception.

## Introduction

Multisensory integration is the process by which information from simultaneously experienced unisensory modalities is fused and forms a single percept (Marks et al., 2018). Investigating the neural networks subserving the multisensory integration could uncover insightful knowledge about human perception pathways. The precise mechanisms underlying multisensory integration remain under investigation; however, current knowledge indicates that it takes place in both lower and higher-order brain structures (Cappe et al., 2012).

The prevailing theory supports that the process accountable for achieving multisensory perception is the convergence of information from different senses on the same neurons, termed multimodal, located in various cortical and subcortical regions (Ghazanfar and Schroeder, 2006; van der Stoep et al., 2017; Merrikhi et al., 2022). Those neurons exhibit distinct integrative properties depending on their location and synaptic architecture. For example, only a minority of cortical bimodal neurons (17%) in monkeys generate a superadditive response, compared to neurons in the Superior Colliculus (55%) and with a substantially lower degree of enhancement (33% vs. 88%). Furthermore, dense and/or axo-somatic synapses are more likely to affect the post-synaptic neuron’s spiking activity than the sparse and/or axo-dendritic ones (Meredith et al., 2012). An additional concept that could support supplementarily the multisensory interplay concerns the mixing of inputs from different cortical areas due to temporal convergence of the incoming information (Fries et al., 2001; Cappe et al., 2012).

Another way to explicate the integration of information across sensory modalities is through the synchronization of neural oscillations (Kayser et al., 2008; Engel et al., 2012; Bauer et al., 2020). This concept is considered to occur through phase reset or phase shifting that exerts a neural oscillation generated in an early or association sensory cortex to another neural oscillation of different localization (Lakatos et al., 2009; Engel et al., 2012). More importantly, the aforementioned theory can interpret multimodal interactions from micro- to large-scale levels. Whether the underlying mechanism of neural coherence is the propagation of the signal through anatomical connectivity or this synchronization could also stem from wireless communication between brain areas, i.e., through the effects of electric field -ephaptic interactions-, and, thus, constitute a mechanism of multisensory integration on its own, remains an open question (Durand, 2019; Depannemaecker et al., 2020).

Up to date, the exact neuronal loci of multisensory integration cannot be identified in humans since the non-invasive electrophysiological (e.g., EEG, MEG) and imaging techniques (e.g., fMRI) used during cognitive tasks are not designed to track the response amplitudes at the single-neuron level. However, through these instrumental methods, the flow of information at the large-scale level, i.e., across brain structures, can be determined by utilizing the metric of effective connectivity. Anatomical studies in non-human primates, along with task-based functional studies in multimodal environments in humans, have established the presence of diverse feedforward, lateral, and feedback connections between and within primary and higher-order sensory cortices (Driver and Spence, 2000; Foxe and Schroeder, 2005; Ghazanfar and Schroeder, 2006; Kayser et al., 2007), as well as subcortical regions (Cappe et al., 2009), reinforcing the scenario of the existence of multisensory neurons throughout multiple brain areas. In parallel, we are moving away from the theory of strict hierarchical organization of the brain, since studies have shown that higher-order cortical areas are activated at about the same time as lower-order areas (Nowak and Bullier, 1997; Schroeder et al., 2001; Merrikhi et al., 2022).

From a cognitive point of view, temporal or spatial correspondence of the external cues is highly important when deciding which stimuli to integrate into a common percept and which to retain as segregated events (Noppeney, 2021). However, an assessment based solely on these stimulus characteristics is insufficient; the stimuli must also cohere in a way that provides logical validity to the common percept. Within the framework of human evaluations, multisensory integration can be studied in conditions where the subjects are exposed simultaneously to multiple stimuli and called upon to respond to them according to some abstract binding rules indicating correspondence of the incoming information (Paraskevopoulos et al., 2021). More specifically, a method of this kind that has been developed involves detecting congruencies and incongruencies between information acquired from different senses. This approach employs the capacity of Mismatch Negativity (MMN) to reflect the brain’s automatic detection of the deviance between the incoming input and the internally generated representation (Garrido et al., 2009; O’Reilly and O’Reilly, 2021; Csépe and Honbolygó, 2024). This pre-attentive error detection, along with the subsequent reallocation of attention, constitutes elements of deviance/error processing (Berti, 2013). An example of a multisensory paradigm, where the detection of congruencies and incongruencies can be implemented, is music notation reading, which combines visual, auditory, and potentially motor information by music learners (Stewart et al., 2003).

The effects of multisensory training on the brain’s neuroplasticity and its supremacy as a learning method over unisensory constitute a topic of perpetual interest in cognitive neuroscience. A notable relevant research work revealed through the computational modeling of an oscillatory neural network, that the use of multiple channels accelerates learning and recall by up to 80% (Rao, 2018). According to a recent electroencephalogram (EEG) study by our group (Paraskevopoulos et al., 2024), uni- and multi-sensory training may induce neuroplastic changes via different mechanisms: multisensory training affects multisensory processing via focal changes in the β band of brain oscillations, while unisensory training increases cross-frequency coupling between θ-β and α-γ frequencies across distributed cortical regions. Interestingly, researchers established at the cellular level the conversion of unisensory neurons to multisensory following cross-modal learning through dopaminergic reinforcement in the species of *Drosophila* (Okray et al., 2023).

Musical learning, a fundamental multisensory training method, has been demonstrated to have notable effects on functional and structural neuroplasticity, as indicated by M/EEG, fMRI, and DTI studies, respectively (Jäncke, 2009). In turn, training-induced plasticity alters how information from different modalities is integrated (Paraskevopoulos and Herholz, 2013). For instance, musicians integrate audiovisual information differently from non-musicians (Schulz et al., 2003; Haslinger et al., 2005; Lee and Noppeney, 2011; Paraskevopoulos et al., 2012), show increased resting state functional connectivity among motor and multisensory cortices (Luo et al., 2012) and exhibit enhanced structural connectivity in regions (Imfeld et al., 2009; Oechslin et al., 2010) such as the corpus callosum (Bengtsson et al., 2005). Even short-term piano training in non-musicians can induce neuroplastic changes in areas including the premotor region, the IPS, Broca’s area, and the inferior parietal region (D’Ausilio et al., 2006; Lahav et al., 2007).

The present work utilizes high-density EEG recordings to model the effective connectome of the cortex during music notation reading in people with no prior musical education and to investigate the network modifications induced by 4 weeks of multisensory or unisensory training. The neuronal activity was captured pre- and post-training during an audiovisual-oddball paradigm, which encompasses combined auditory and visual stimuli in congruency or incongruency, depending on whether they obey the rule “the higher the pitch, the higher the position of the disk.” At the same time, unisensory mismatches that pertain to the color and the timbre of the note are embedded in the experiment, targeting the visual and the auditory system, respectively. The hypothesis is that multisensory training will have a significantly different impact on the effective connectivity of the cortical networks subserving the audiovisual, visual, and auditory processing compared to unisensory, as regards the number of modified connections and the regions involved in the connectivity changes.

## Materials and methods

### Subjects

In this study, a total of thirty participants were recruited. They were divided into two groups: the first group (MusicPlast group) comprised 15 subjects that received multisensory/musical training (age range: 18–35; mean age: 26.53; SD: 3.14; 5 males) and the second group (UniPlast group) consisted of 15 subjects that received unisensory training, (age range: 18–35; mean age: 28.67; SD: 5.39; 5 male). The required number of participants was determined through an *a priori* power analysis conducted using G*Power version 3.1.9.7. The analysis was based on effect size estimates derived from a previous study employing a similar paradigm and analysis (Paraskevopoulos et al., 2015). Specifically, the power analysis targeted an *F*-test for a repeated-measures ANOVA with a within-between interaction, using an effect size (f) of 0.359 calculated from the interaction effect on network density in the earlier study. We set the significance level at α = 0.05, the desired statistical power at 0.95, with 2 groups and 4 measurement points (i.e., combinations of condition and time). Based on these parameters, a total sample size of 30 participants (15 per group) was determined to be sufficient, yielding an actual power of 0.998. None of the subjects had received musical education before the study, apart from the mandatory lessons in primary and high school. Furthermore, all participants were right-handed based on the Edinburgh Handedness Inventory (Oldfield, 1971), exhibited normal auditory capacity, normal or corrected-to-normal vision, and did not mention any neurological condition, mental health disorder, prior brain injury, or intake of central nervous drugs, that could compromise the reliability of the results of the study. Informed consent in written form was obtained from all participants before their participation in the study. The study protocol was approved by the Aristotle University of Thessaloniki Research Ethics Committee. Participants were recruited as part of MusicPlast, a randomized controlled trial registered at ClinicalTrials.gov under identifier code NCT03786185. All participants completed the 4-weeks training intervention (unisensory or multisensory) and the pre- and post-training evaluation.

### Experimental procedures

#### Stimuli

During the experiment, proposed initially by Paraskevopoulos et al. (2012), the subjects received audio-visual information through sequences of 5 concurrent images and sounds. The images were composed of five white horizontal lines on a black background and a blue circle placed in the middle of the horizontal direction and in one of the four spaces between the lines. The auditory stimuli corresponded to tones of a concrete pitch. Combinations of four different sinusoidal tones were used to create the five-tone melodies (F5, 698.46 Hz; A5, 880.46 Hz; C6, 1046.50 Hz; and E6, 1318.51 Hz), with the first tone of all melodies being C5. The tones had a duration of 400 and 10 ms rise and decay time (44,100 kHz, 16 bit). Each melody had an interstimulus interval of 500 ms between each tone, resulting in a total duration of 4 s for each melody. For each tone played, the blue circle appeared at the appropriate location at the same time and for the same duration as the tone.

**FIGURE 1:**
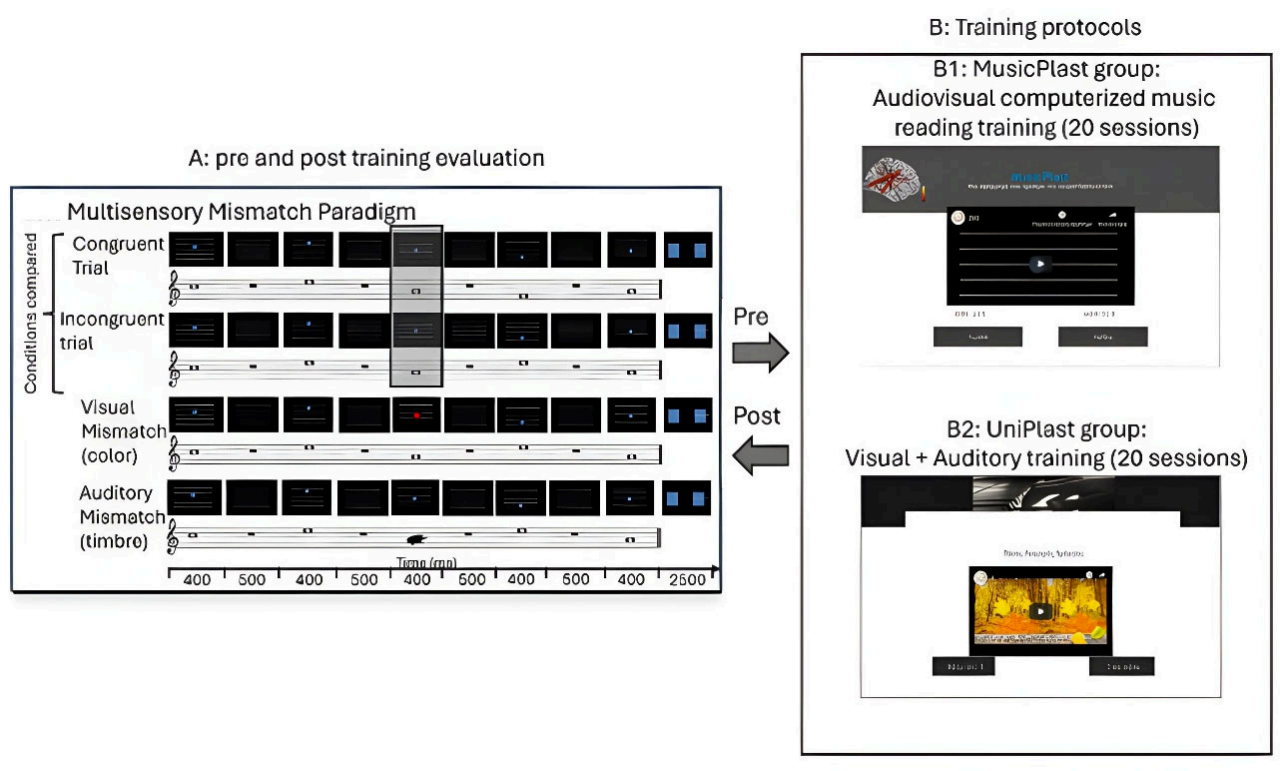
Illustration of the experimental procedure. **(A)** Pre and post-training evaluation through high-density EEG recordings during an audiovisual oddball paradigm. **(B1)** MusicPlast training: audiovisual (multisensory) training via a computerized music reading regime. **(B2)** UniPlast training: simultaneous auditory and visual training that targeted each modality independently; hence, unisensory rather than multisensory integration processes were employed.

There were four different conditions embedded in the experiment, depending on the accordance within and between the visual and auditory stimuli. The audio-visual stimuli could be congruent or incongruent depending on whether they obeyed the rule “the higher the pitch, the higher the position of the disk” or not. This principle resembles the well-established notion of crossmodal correspondence (Spence, 2011; Parise and Spence, 2012); however, in the present paradigm, it is implemented as a rule-based, sequential mapping akin to musical notation (movable C), where relative transformations rather than absolute feature associations determine congruency. Moreover, there could be a deviance of visual or auditory stimulus, suggested by a difference in the color of the circle or the timbre of the tone, respectively. Specifically, the auditory (timbre) deviant stimuli were prepared by replacing one of the tones with another of the same frequency, generated using a sawtooth waveform, and filtered with a low-pass filter set at 5000 Hz. For the visual (color) deviant stimuli, the blue circle of one of the images was replaced with a red circle, creating a mismatch in terms of the visual input. In both cases of unisensory mismatches, there was no violation of the rule of congruency between sound and image, since the pitch height and the position of the circle coincided. Additionally, the deviance was never in the first tone–image pair but was equally likely to appear in each of the other four places. Therefore, in total, there were four conditions of stimuli: the audiovisual congruent, the audiovisual incongruent, the visual mismatch, and the auditory mismatch (Figure 1A). For each of the four video categories, eight distinct melodies were prepared.

#### Apparatus

The brain activity induced by the multisensory stimuli was recorded by a Nihon-Kohden 128-channel EEG system with an active (actiCAP128, Brain Products) and a passive (R-Net, Brain Products) electrode cap. The caps were evenly distributed among the groups, and each subject consistently wore the same cap for both pre-training and post-training measurements. The data acquisition was performed with a sampling rate of 1000 Hz and took place in a controlled environment within an electrically shielded and acoustically insulated room. To ensure optimal signal quality, the impedance of the electrodes used in the ActiCAP system was maintained below 5 kΩ, and that of those used in the R-Net below 20 kΩ. For the auditory stimulation, participants wore closed-type headphones (Phillips SHL3260) through which sound was presented at a level 60 dB higher than their individual hearing threshold. Regarding the visual stimulation, a flat-panel display was positioned approximately 110 cm away from each participant. The display had a refresh rate of 60 Hz and a spatial resolution of 1280 × 768 pixels. The visual stimulus covered a viewing angle ranging from −1.15° to 1.15° vertically and from −3.86° to 3.86° horizontally. To ensure the subjects’ attentiveness and cooperation during the experiment, video monitoring was employed to verify their alertness and compliance.

#### Design

Videos of all four conditions were presented via Presentation software (Version 18.0, Neurobehavioral Systems, Inc., Berkeley, CA)^1^ to the subjects, who subsequently had to respond to the stimuli according to their accordance via button press. More specifically, before the beginning of the EEG recordings, the participants were provided with instructions and tried out a video example from each category. In total, 512 videos were presented to the participants (128 videos of each category), separated into 4 blocks, with each block lasting ∼14.5 min with short breaks in between. Videos were randomly mixed together, and after the presentation of each video, the participants had 2.5 s to respond if the video was congruent or incongruent, using two buttons in their right hand, and if there was a tone sounding differently than all others or a circle of a different color, using two buttons in their left hand. Moreover, on the screen, it was shown which button corresponds to each answer.

#### Multisensory training

The first group, named MusicPlast, received multisensory training through an online platform^2^ that is accessible by desktop or mobile devices and implements the presentation of videos in a serious game environment. The web-based platform was initially developed for the study of Paraskevopoulos et al. (2021), using the software Adobe Captivate 2017.^3^ In contrast to the experimental procedure, the training involved only two out of the four categories of stimuli: the audiovisual congruent and the audiovisual incongruent. The two conditions were prepared in the same manner as those of the experiment, with the exception that the melodies weren’t simple sequences of tones but short segments of 40 famous Greek songs performed with piano. As regards the incongruent videos, only for one of the tones included, the position of the disk violated the rule “the higher the pitch of the tone, the higher the position of the disk,” and the mismatch was never presented in the first or the last tone of the melody (Figure 1B1).

Furthermore, 3 additional levels of difficulty were created: In the second level, background noise (white noise) was added to the sound of the videos, at a volume 6 decibels lower than that of the piano tones. The third level included a visual distortion of the videos through the application of grain to the video medium, set at an opacity of 75%. The fourth and final level, analogous to the third, involved both auditory and visual distortions, with white noise having higher intensity (the same decibels as piano tones).

The duration of each video spanned from 4.92 to 8.25 s (*M* = 6.89, SD = 8.42), while the number of tones incorporated in each video ranged from 8 to 16 (*M* = 13, SD = 2). 80 videos were generated for each level of difficulty (40 congruent and 40 incongruent) and randomly distributed in 5 sets (sessions) of 55 videos each, with each session lasting about 20 min. Thus, the platform included 20 sessions in total. Over the course of 4 weeks, participants were required to complete all 20 training sessions, finishing a difficulty level (5 sessions) per week. Utilizing their personal devices, including desktop and mobile devices, participants were directed to access the e-learning platform’s webpage.

After the presentation of each video, the participants had to respond through a button press whether there was a violation of the above-mentioned rule or not. Subsequent to the button press, a message on the screen, lasting for 3 s, informed them about the correctness of their answer. At the end of each practice session, the website displayed their score as a percentage of accurate responses. Simultaneously, a box popped up and requested for their special code, transferring their data to the training website.

#### Unisensory training

The second group, named UniPlast, received unisensory training through the same online platform,^4^ accessible by desktop or mobile devices. The training procedure triggered concurrently both the visual and auditory domains, but targeted each one independently, indicating that multisensory processes weren’t employed. Specifically, in the control task, the subjects were exposed to a video containing 2 similar images that could differ or not by some details and were asked to spot the differences (visual training); at the same time, they listened to a familiar musical piece that could be modified or not to include a dissonant part. At the end of the video, they were called to respond to whether the music contained a dissonant part (auditory training) and how many differences they spotted in the image (Figure 1B2). Immediately after their response, the subjects received a feedback for 3 s on the correctness of their choice, and the procedure moved to the next video. Each video lasted for 4 min, and each session included 4 such videos, compiling a training session of equal length to the MusicPlast one.

Subjects had to perform 20 sessions of the training, which following the prototype of the MusicPlast one, had 4 levels of difficulty, defined by the amount of visual or auditory noise added to the video to additionally train the ability to discriminate the (auditory or visual) object from noise (first level has no noise, second has auditory noise, third auditory and visual noise and fourth one auditory and visual, but of increased noise intensity). At the end of each session, the platform presented their score as a percentage of correct responses, and a pop-up window prompted them to provide their participant code and send the data to the e-learning platform.

Importantly, the control training employed divided attention (the trainee had to focus on both the visual and the auditory task at the same time), and hence greater attentional resources were expected to be recruited. This differentiation across the two trainings managed to be balanced by their overall structure, which included a slower stimulus presentation rate in the UniPlast vs. the MusicPlast training (4 videos of 4 min each in the control training, while 55 videos of ∼ 5 s each in the MusicPlast training).

### EEG data analysis

#### EEG preprocessing

EEG data were preprocessed using Brainstorm, an open-source application that performs analysis of brain recordings.^5^ The recorded data were segregated into epochs of 4 s around the onset of the stimulus, including a pre-stimulus interval of 2 s, and imported into the database. Afterward, artifacts due to eye blinks or any other bad segments were removed by an automatic artifact detection algorithm embedded in Brainstorm. The aforementioned algorithm performs Signal-Space Projection, which removes artifacts with consistent spatial patterns, such as eye blinks or muscle activity, by computing a projection matrix that filters the signal (Vosskuhl et al., 2020). The projection matrix is the inverse matrix of a cross-covariance matrix between the reference signals (EOG, EMG) and the EEG signal (Uusitalo and Ilmoniemi, 1997). To avoid introducing distortions into the effective connectivity (Granger Causality) estimates, notch and band-pass filters were omitted (Bressler and Seth, 2011).

Subsequently, four different event conditions were defined: standard, audiovisual incongruent, auditory deviant, and visual deviant. Each epoch was synchronized to the last stimulus of each stimulus pattern and, following, the DC offset was removed by calculating the average signal of each channel at the time window [−400, −1] ms prior to stimulus onset and subtracting it from the signal of each channel at the full epoch interval [−2, 2] s.

#### EEG source analysis

EEG source analysis was performed using sLORETA, implemented in Brainstorm (see text footnote 5), for the neural responses of each subject, for each time-point (pre or post), for each stimulus category (standard, audiovisual incongruent, visual, and auditory deviant), and for each single trial. The resulting time series were assigned to the 360 regions included in the HCP-MMP1.0 atlas (Glasser et al., 2016), by performing Principal Component Analysis decomposition (PCA) on the signals of each parcel and opting for the first mode. The selection of this atlas was made to achieve a compromise between two important factors. Specifically, it aimed to offer an adequate level of functional and interpretational precision in reconstructing cortical activity time series through a multi-modal parcellation approach, while it intended to significantly decrease the number of reconstructed sources (Paraskevopoulos et al., 2021). In this manner, a total of 360 source time-series were extracted.

#### Effective connectivity analysis

Bivariate Granger Causality was employed as a robust metric of effective connectivity that has been extensively used and tested in cortical network studies (Formaggio et al., 2021; Wang et al., 2024). The method, implemented in Brainstorm (see text footnote 5), was applied to measure the effective connectivity between the 360 regions of the HCP-MMP1.0 atlas for each trial of each condition (auditory deviant, visual deviant, audiovisual incongruent, and standard), subject, and time-point. In total 17037 trials were exported to estimate an equal number of 360 × 360 adjacency matrices filled with effective connectivity coefficients. The directed, and hence asymmetric, adjacency matrices of the different trials of each condition of each subject of each time point (pre-post) were then averaged, resulting in one connectivity matrix per stimulus category per subject per time-point. Thus, 30 (subjects) × 2 (time-points) × 4 (categories) = 240 adjacency matrices were generated, which were later unified in a 360 × 360 × 240 3-D array (tensor).

#### Statistical analysis

The Network-Based Statistic (NBS) toolbox in MATLAB was used to identify the statistically significant alterations of connectivity patterns under the statistical framework of the Generalized Linear Model (GLM). Initially, the 360 × 360 × 240 tensor with the effective connectivity coefficients was extracted from Brainstorm, and three types of statistical comparisons took place via permutation (non-parametric) tests.

More specifically, the first analysis included a permutation test of one factor (Condition: Standard and Deviant) for each type of deviance (audiovisual, visual, auditory) for all of the subjects in the « Pre- » time-point. This analysis aims to reveal the connections that subserve the error processing in the audiovisual, visual, and auditory modality in people without previous musical education. The second analysis implemented a 2 × 2 Generalized Linear Mixed Model (Condition: standard and deviant, Time: pre- and post-training) for each type of deviance (audiovisual, visual, auditory) and each group (UniPlast and MusicPlast). Therefore, by comparing the cortical networks of the standard versus the deviant condition and before versus after each type of training within the corresponding group, this analysis showcases the error processing-modality-specific neuroplasticity, induced by the unisensory and multisensory training, respectively. Lastly, a third analysis was performed, which implemented a 2 × 2 × 2 General Linear Mixed Model (Condition: standard and deviant, Time: pre- and post-training, and Group: UniPlast and MusicPlast) for each type of deviance (audiovisual, visual, auditory) in an effort to indicate directly the difference between the neuroplastic effects of the two trainings. The computation of the modality-specific networks depicting the differences between two distinct conditions (e.g., standard versus deviant) or additionally between two time points (e.g., pre- versus post-training) or two different groups (e.g., UniPlast versus MusicPlast) has the advantage of examining issues exclusively related to the process of information integration, as attention and other cognitive functions are interchangeable among the different sensory conditions and thus don’t affect the interpretation of sensory interactions (Calvert and Thesen, 2004).

For all the analyses, 10.000 permutations were used, while the significance level was set to *P* < 0.001 corrected for multiple comparisons via false discovery rate (FDR) correction. In the end, the visualization of the significantly altered effective connectivity networks was performed using BrainNet Viewer, and thus, graphs with nodes and color-weighted edges, indicative of the F-statistic value of each statistical comparison, were generated. For the description of the brain regions involved in the statistically significant networks, the HCP-MMP1.0 atlas nomenclature system was used (Glasser et al., 2016), with each label corresponding to a specific parcellation.

## Results

### Behavioral results

To assess the impact of training on audiovisual processing, we compared participants’ behavioral performance on the discriminability index before and after the intervention. Statistical analysis revealed a significant Group × Time interaction [F(1,28) = 4.635; *p* = 0.042; η^2^ = 0.168], demonstrating that multisensory and unisensory training had differential effects on participants’ ability to detect audiovisual incongruencies. Specifically, the MusicPlast group showed a clear improvement in discrimination accuracy (pre-training: *M* = 2.22, SD = 0.44; post-training: *M* = 2.89, SD = 0.82), whereas the UniPlast group did not exhibit such gains (pre-training: *M* = 2.26, SD = 0.49; post-training: *M* = 2.17, SD = 0.82). Neither the main effect of Group nor of Time reached significance.

### EEG results

#### Audiovisual incongruency identification prior to any training

**FIGURE 2:**
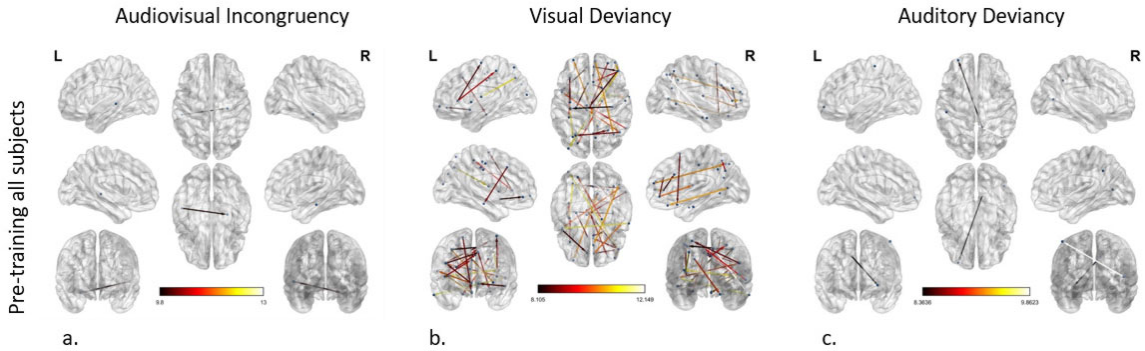
Effective connectivity networks prior to training activated by **(a)** audiovisual incongruency detection, **(b)** visual deviancy detection, and **(c)** auditory deviancy detection. Maps of the significant networks derived from the statistical comparison between the standard and the deviant condition for each modality are presented. Significance level for all three analyses was set to *P* < 0.001 corrected for multiple comparisons via false discovery rate (FDR) correction, using 10,000 permutations. The color scale used for the depiction of the edges’ significance indicates the *F*-value range, with a threshold value set at 8.

To model the network differences between the standard and the deviant condition, i.e., the connectome subserving error processing in the audiovisual modality of all subjects before training, we applied the statistical threshold of *p* < 0.001 corrected for multiple comparisons. To further enhance interpretability and focus on the most robust effects within this already significant set, we subsequently applied an additional *F*-value threshold of *F* ≥ 8. The analysis revealed an altered connection between the left dorsal Superior Temporal Sulcus (STSd) and the right Hippocampus (H) (Figure 2a).

#### Visual deviancy identification prior to any training

The modeling of the network subserving error processing in the visual modality of all subjects before training uncovered cortical reorganization in plenty of connections (nodes: 40; edges: 24), setting the *p*-value < 0.001 and the additional *F*-value > 8. As indicated by node strength, the region with the greatest role in the network was the right IFSa of the inferior frontal cortex, participating in 4 out of 24 connections. The 5 most significant connections (*F*-value > 10.57) were detected as follows: (a, b) from the right IFSa to the right anterior area 9-46v (a9-46v) in the dorsolateral prefrontal cortex and to the left area V3CD of the MT + complex, (c, d) from the right anterior area TE1 (TE1a) of the lateral temporal cortex to the right medial area 7P (7Pm) of the superior parietal cortex and to the left Frontal Opercular Area 1 (FOP1) in the posterior opercular cortex, and (e) from the left Intraparietal Area 1 (IP1), which is located in the inferior parietal cortex, to the left Insular granular complex (lg) of the Insular Cortex (IC) (Figure 2b).

#### Auditory deviancy identification prior to any training

Comparing the cortical networks in all subjects between the standard and the deviant auditory condition before the training, two connections were found to be significantly different, setting the *p*-value < 0.001 and the additional *F*-value > 8. These connections arise from the right area 31pd in the posterior cingulate cortex (PCC) and the right Medial Superior Temporal Area (MST) of the dorsal visual stream and point to the left anterior area 10p (a10p) in the orbital prefrontal cortex and the left Primary Somatosensory Cortex (area 1), respectively (Figure 2c).

#### Audiovisual incongruency identification before versus after multisensory training

**FIGURE 3:**
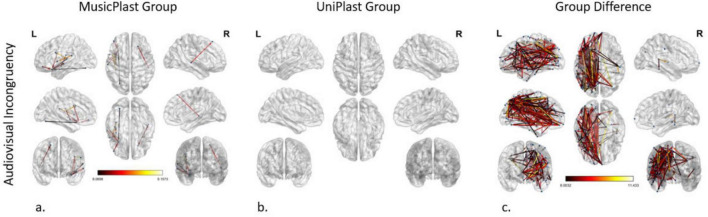
Network modifications supporting audiovisual incongruency detection induced by **(a)** multisensory training in MusicPlast Group and **(b)** unisensory training in UniPlast Group. **(c)** Difference in network modifications supporting audiovisual incongruency detection between the MusicPlast and the UniPlast Group. Statistical parametric maps of the significant networks for the 2 × 2 interaction of condition (congruent vs. incongruent stimuli) × time point (Pre- vs. Post-training) for each Group **(a,b)**, as well as for the triple interaction of condition (congruent vs. incongruent stimuli) × time point (Pre- vs. Post-training) × Group (MusicPlast vs. UniPlast) **(c)** are presented. The significance level was set to *P* < 0.001 corrected for multiple comparisons via false discovery rate (FDR) correction, using 10,000 permutations. The analysis of the UniPlast Group failed to display any statistically significant network reorganization, even after increasing the *p*-value threshold to 0.05. The color scale used for the depiction of the edges’ significance indicates the *F*-value range, with a threshold value set at 8.

The corresponding *F*-test for the effect of the multisensory (MusicPlast group) training in the audiovisual modality identified 11 statistically significant connections between 7 nodes (*p*-value < 0.001; *F*-value > 8), with 5 of them including the left area 47 s, located in the orbital prefrontal cortex. Nevertheless, the most significant reorganization (*F*-value > 8.63) concerned the following connections: (a, b) from the left area IFJp in the inferior frontal cortex and the left Posterior Insular area (PoI1) in the insular cortex to the left ventral area 6 (6v) in the premotor cortex, (c) from the left RetroInsular Cortex (RI) in the early auditory cortex to the left FOP1 and (d) from the left Primary Auditory Cortex (A1) to the left area IFJa in the Inferior Frontal Cortex (Figure 3a).

#### Audiovisual incongruency identification before versus after unisensory training

The analysis of the unisensory training’s effect on the UniPlast Group’s capacity for error processing in the audiovisual modality failed to display any statistically significant network reorganization, even after increasing the *p*-value threshold to 0.05 (Figure 3b).

#### Audiovisual incongruency identification, difference of the trainings’ effects

The triple interaction of the General Linear Model representing the difference between the effects of the two types of training in the audiovisual modality using a *p*-value < 0.001 and an additional *F*-value > 8 identified a network of great density (nodes: 116; edges: 206). Importantly, the 5 most statistically significant connections that were depicted (*F*-value > 10.84) are the following: (a) from the left area 10v of the Anterior Cingulate cortex (ACC) to the left Primary Motor Cortex (area 4), (b, c) from the left area 52 of the Early Auditory Cortex to the left Superior 6-8 Transitional area (s6-8) in the dorsolateral prefrontal cortex and the left anterior area 6 m (6ma) in the paracentral lobule, (d) from the left area 8C in the dorsolateral prefrontal cortex to the left area 7PC in the superior parietal cortex, and (e) from the left Medial Belt Complex (MBelt) in the Early Auditory Cortex to the left IFJa (Figure 3c).

#### Visual deviancy identification before versus after multisensory training

**FIGURE 4:**
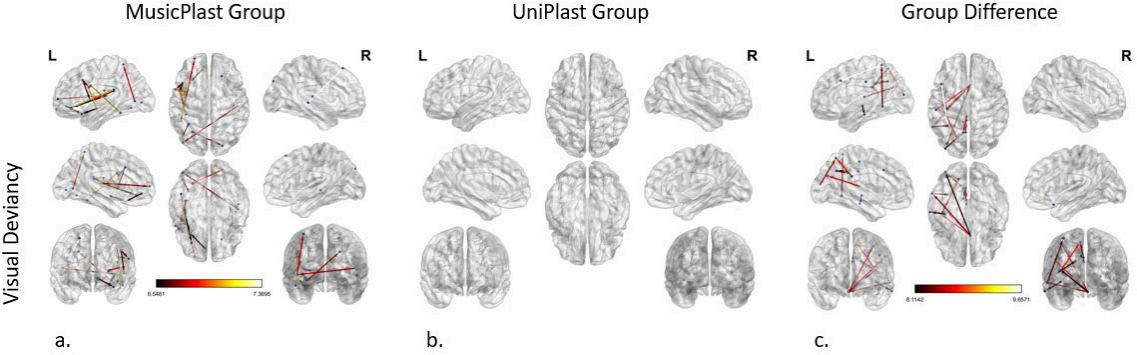
Network modifications supporting visual deviancy detection induced by **(a)** multisensory training in MusicPlast Group and **(b)** unisensory training in UniPlast Group. **(c)** Difference in network modifications supporting visual deviancy detection between the MusicPlast and the UniPlast Group. Statistical parametric maps of the significant networks for the 2 × 2 interaction of condition (standard vs. deviant stimulus) × time point (Pre- vs. Post-training) for each Group **(a,b)**, as well as for the triple interaction of condition (standard vs. deviant stimulus) × time point (Pre- vs. Post-training) × Group (MusicPlast vs. UniPlast) **(c)** are presented. The significance level was set to *P* < 0.001 corrected for multiple comparisons via false discovery rate (FDR) correction, using 10,000 permutations. The analysis of the UniPlast Group failed to display any statistically significant network reorganization, even after increasing the *p*-value threshold to 0.05. The color scale used for the depiction of the edges’ significance indicates the *F*-value range. The threshold was set initially at 8 for the analyses with statistically significant results, however, it was lowered to 6.5 for the MusicPlast Group analysis because, until then, no connections had been detected.

The modeling of the network modifications supporting the visual deviancies in the MusicPlast Group, using a *p*-value threshold of 0.001 and an additional *F*-value threshold of 6.5, managed to identify 16 altered connections between 22 regions, mainly of the prefrontal cortex. The *F*-value threshold of 6.5 -slightly less stringent than the *F* ≥ 8 used in other analyses- was selected as a compromise, since omitting the *F*-value threshold produced an unmanageably large number of connections, whereas the initial cutoff of *F* ≥ 8 yielded none. This adjustment allowed for a feasible number of connections to be visualized while ensuring statistical validity. The left PoI1 was recognized as the node with the maximum strength, sharing 3 out of 16 connections. The 5 most statistically significant connections (*F*-value > 6.95) were depicted as follows: (a) from the left area 47 m in the orbital prefrontal cortex to the left area PFcm in the early auditory cortex, (b) from the left IFJp to the left posterior area TE2 (TE2p) of the lateral temporal cortex, (c) from the right dorsoposterior Superior Temporal Sulcus (STSdp) to the right s6-8 and (d, e) from the left PoI1 to the left 6v and the left OP2-3 area of the posterior opercular cortex (Figure 4a).

#### Visual deviancy identification before versus after unisensory training

The analysis of the unisensory training’s effect on the UniPlast Group’s capacity for error processing in the visual modality also failed to display any statistically significant network reorganization, even after increasing the *p*-value threshold to 0.05 (Figure 4b).

#### Visual deviancy identification, difference of the trainings’ effects

The statistical analysis of the effect difference between the two types of training in detecting visual deviances identified a network of 16 nodes and 12 edges, applying a threshold of *p*-value at 0.001 and an additional *F*-value at 8. Interestingly, region 25 in the ACC of the right hemisphere shares 4 out of the total 12 connections with areas from all four lobes, displaying the maximum node strength. The most statistically significant connection (*F*-value = 9.66) links the left 8C to the left 7PC (Figure 4c).

#### Auditory deviancy identification before versus after multisensory training

**FIGURE 5:**
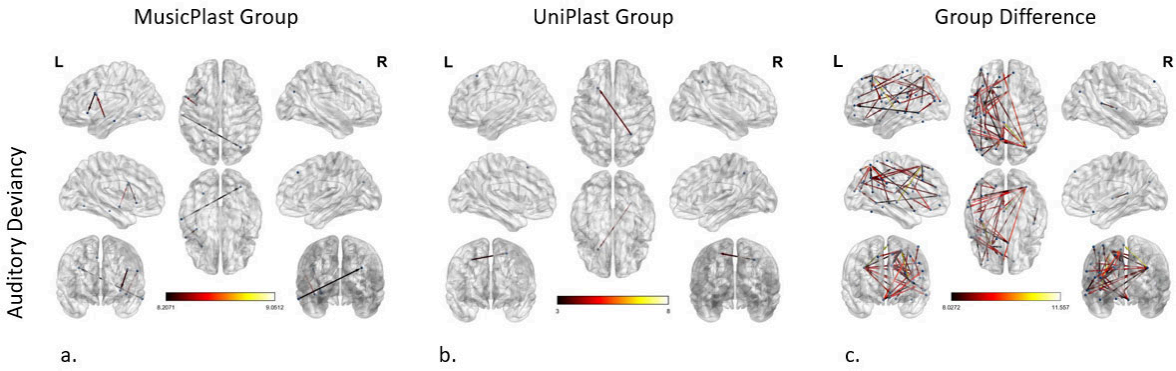
Network modifications supporting auditory deviancy detection induced by **(a)** multisensory training in MusicPlast Group and **(b)** unisensory training in UniPlast Group. **(c)** Difference in network modifications supporting auditory deviancy detection between the MusicPlast and the UniPlast Group. Statistical parametric maps of the significant networks for the 2 × 2 interaction of condition (standard vs. deviant stimulus) × time point (Pre- vs. Post-training) for each Group **(a,b)**, as well as for the triple interaction of condition (standard vs. deviant stimulus) × time point (Pre- vs. Post-training) × Group (MusicPlast vs. UniPlast) **(c)** are presented. The significance level was set for the analysis of the MusicPlast Group and the Group Difference to *P* < 0.001, corrected for multiple comparisons via false discovery rate (FDR) correction, using 10,000 permutations. The analysis of the UniPlast Group failed to display any statistically significant network reorganization with *p*-value < 0.001, and therefore we increased the *p*-value threshold to 0.05 to identify any modifications. The color scale used for the depiction of the edges’ significance indicates the *F*-value range, with a threshold value set at 8.

Statistical comparisons (2 × 2 mixed General Linear Model) depicting the effect of the multisensory training on the network supporting the identification of the deviances of audiovisual nature (*p*-value < 0.001 and an *F*-value > 8) demonstrated the following connectivity alterations (nodes: 8; edges: 4): (a) from the left IFJa to the left Anterior Ventral Insular area (AVI) in the insular cortex, (b) from the left PoI1 to the left 6v, (c) from the left middle TE1 area (TE1m) in the lateral temporal cortex to the right Intraparietal area 0 (IP0) in the inferior parietal cortex, and (d) from the Ventromedial Visual area 3 (VMV3) of the ventral visual stream to the left 8BM area in the ACC, connection that displayed the maximum *F*-value (9.05) (Figure 5a).

#### Auditory deviancy identification before versus after unisensory training

The corresponding *F*-test on the effect of the unisensory training on detecting the auditory irregularities managed to identify one modified connection, and only after increasing the *p*-value to 0.05. Specifically, this connection springs from the right dorsal Lateral Intraparietal area (LIPd) and points to the left s6-8 (Figure 5b).

#### Auditory deviancy identification, difference of the trainings’ effects

Additional statistical comparisons (General Mixed Model of 3 factors and setting the *p*-value > 0.001 and the additional *F*-value > 8) identified an especially dense network of 46 edges and 49 nodes. Nevertheless, the increase of the *F*-value to 9, reduced the complexity of the network (edges: 16, nodes: 21). The area with the maximum node strength (5 out of 16 edges) was the right IP0 with connections to the frontal, parietal and temporal lobes. The 3 most significantly modified connections (*F*-value > 10.29) were the connections: (a) from the right Lateral Belt (LBelt) in the early auditory cortex to the left area 5 m in the paracentral lobule, (b) from the left 8C to the left 7PC, and (c) from the left area 52 to the left s6-8 (Figure 5c).

## Discussion

In this study, we investigated the neuroplastic effects of multisensory and unisensory training by scrutinizing the network reorganization, accountable for error processing, in the audiovisual, visual, and auditory modality after 4 weeks of each training. Additionally, effective connectivity networks that subserve mismatch detection in all three modalities among all participants prior to their training were modeled. Calculating modality-specific networks that illustrate the differences between two conditions (e.g., standard versus deviant) provides the benefit of focusing on information integration processes alone, since the rest of the cognitive functions, such as memory or attention, remain constant across the two conditions, and thus, their effect is not depicted on the ensuing networks.

The outcomes of the first statistical analysis, comparing the standard and deviant conditions before any type of training, revealed that individuals without prior musical education exhibit a higher density of cortical connections associated with the detection of visual mismatches compared to the network recognizing auditory or audiovisual deviances. Nevertheless, this observation may reflect the specific cues included in the experimental procedure, such as the varying degrees of stimulus strength across each modality. Color, for instance, could constitute a more powerful stimulus for the visual system than timbre is for the auditory system. Another explanation could be that people without musical education weigh the visual information more than the auditory (Paraskevopoulos et al., 2015). The STS was shown to have a significant function during the integration of audiovisual incongruencies, an outcome that comes in accordance with a variety of findings in the existing literature (Hikosaka et al., 1988; Barraclough et al., 2005; Cappe et al., 2009). Concerning the visual modality, the right IFSa, a short-term-memory-related inferior frontal area (Rolls et al., 2023), was found to be a principal node for detecting visual mismatches, while its effective connectivity to VC3D of the MT + complex opposes the hypothesis of a strict hierarchical model, even within the visual system. This finding complies with evidence that association areas can affect information processing in unisensory systems via processes of down-stream signaling (Skirzewski et al., 2022).

As regards the impact of the multisensory training on the MusicPlast group’s capacity to process information, the identified network demonstrated considerable reconfiguration in all three modalities (audiovisual, visual, and auditory). A greater degree of neuroplasticity was presented throughout the integration of audiovisual incongruencies. More precisely, the left region 47 s of the orbital prefrontal cortex was found to be a key structure of reorganization associated with tasks of detecting cross-modal deviances. In literature, area 47 s has been related to the processing of linguistic syntax, temporal coherence in music, and statistical learning (Levitin and Menon, 2003; Williams, 2020; Paraskevopoulos et al., 2022). The multisensory-induced neuroplastic changes related to the integration of visual and auditory stimuli encompassed mainly connections between higher-order cortical areas however also links from lower to higher-order cortical areas or exclusively between lower-order cortical areas were detected, indicating both top-down and bottom-up processes affecting unisensory information integration.

The corresponding analysis of the Condition x Time interaction in the UniPlast group, which underwent unisensory training, failed to identify any statistically significant difference in the connections serving error processing among the audiovisual and visual modalities. Concerning the detection of irregularities of auditory nature, the statistical comparison before and after training revealed only one altered connection, also within higher-order association areas (LIPd R, s6-8 L). Nevertheless, the lack of neuroplasticity within the visual modality may be attributed to the fact that detecting small cue differences between two similar pictures during unisensory learning may exert a smaller Mismatch Negativity Response compared to the effect of color change in the same picture during multisensory training.

It is not surprising that multisensory training can be more effective than unisensory in inducing neuroplasticity related to multisensory or even unisensory tasks. A similar multifeatured paradigm implicated in another study by Paraskevopoulos et al. (2014) indicated that multisensory training contributes to a greater response during post-training audiovisual tasks in comparison to unisensory. Likewise, as regards the performance on unisensory tasks, various studies have agreed on the superiority of concurrent audiovisual exposure as a learning approach when compared to training with auditory or visual cues alone (Pekkola et al., 2005; Lappe et al., 2008, 2011; Kayser et al., 2009; Hoefer et al., 2013). The findings are logical if we consider that when a higher-level process is modified, it directly impacts lower-level processes through feedback loops. However, when a lower-level process is altered, its effects may be too specialized to automatically influence higher-level processes (Proulx et al., 2012). As a result, training involving multiple senses influences the processing of individual sensory inputs, but training focused on a single sense cannot influence the overall functioning of the multisensory processing system. Therefore, the cognitive hierarchy governing the integration of these processes can serve as a valuable guide for selecting the appropriate training method when targeting specific outcomes.

The analysis of the three-way interaction of Group × Condition × Time point confirmed that the effects of the two trainings are significantly different in all three modalities, especially with regard to the audiovisual. The greatest reorganization dissimilarity was detected around the right 25 area of the ACC for the visual modality, and around the right IP0 for the auditory modality. The discriminability evaluation similarly verified the increased learning effect of the multisensory training on the audiovisual processing.

Overall, among the regions with the most prominent role in our results about multisensory-induced neuroplasticity were found to be the left 6v, which is located in the caudal part of the middle frontal gyrus (MFG), the left 8C also in the posterior part of MFG, the left PoI1 situated in the posterior long gyrus of the insula, and the left IFJa lying in the posterior portion of the inferior frontal sulcus (IFS), near the upper border of inferior frontal gyrus (IFG). Several recent research works have showcased the role of the MFG and IFG as central nodes of the multisensory networks (Li et al., 2020; Junker et al., 2021). Using an almost identical multifeatured paradigm, Paraskevopoulos et al. pinpointed the importance of IFG (Paraskevopoulos et al., 2014, 2022) as a key hub that underwent modifications due to multisensory learning. Correspondingly, the insula, once considered a peripheral element within the existing literature, has gained substantial recognition as a pivotal node in the process of multisensory integration (Renier et al., 2009; Chen et al., 2014). Notably, a recent meta-analysis that included 49 studies characterized the regions: left IFG, bilateral superior temporal gyrus (STG), middle temporal gyrus (MTG), thalamus, and right insula as significant neural correlates serving multisensory integration (Zhe et al., 2021). To delve further, it is claimed that these regions comprise different functional roles, with conflict-processing brain regions such as the insula and the IFS facilitating the integration of incongruent information (Scheliga et al., 2022).

The findings of our study revealed a notable left lateralization within reorganized regions subserving error processing in all three modalities. As regards the auditory MMN, this outcome comes in accordance with new evidence supporting the left hemispheric prevalence during semantic interpretation of lexical tones (Wang et al., 2021) instead of right hemispheric dominance during pure tone paradigms (Garrido et al., 2009). It seems that the presence of an auditory deviation while listening to a possibly familiar melody requires more auditory cognitive processing than non-semantic acoustic cues analysis resources.

Whereas previous research has proposed analogous occurrences in the aforementioned brain regions incited by multisensory experience, our study quantified these reorganizational processes through the modeling of effective connectivity networks. Our recent study (Paraskevopoulos et al., 2024), which uncovered distinct neuroplastic mechanisms in uni- and multisensory training affecting cross-frequency and oscillatory processes related to multisensory perception, also provided evidence for the automatic transferability of the multisensory learning effect. Specifically, when these neural findings were integrated with behavioral and cognitive data, they demonstrated the superiority of multisensory learning in improving multisensory processing while at the same time enhancing general cognitive processes. The present study not only reaffirms the potency of multisensory learning but also highlights its superior efficacy compared to the unisensory domain, even within tasks that rely on unisensory neuronal processes.

Portraying the differential impact of multisensory and unisensory training on the brain’s functional connectivity could elucidate their potential role in rehabilitation and educational strategies. Rehabilitation procedures that utilize complex, multisensory training tasks have gained increasing interest in recent years (Paraskevopoulos and Herholz, 2013; Tortora et al., 2024), with musical training being one of the most studied types of interventions in clinical conditions associated with synaptic disruptions and brain atrophy, such as stroke, Parkinson’s Disease, mild cognitive impairment (MCI), and Alzheimer’s disease. Analogously, musical training could be implemented in the intervention plan for language and reading impairments, such as dyslexia, as it seems that musical expertise acts similarly to linguistic tasks (Musacchia et al., 2007; Paraskevopoulos and Herholz, 2013) and musical ability is positively correlated with linguistic and other cognitive or executive functions (Jaschke et al., 2018; Linnavalli et al., 2018; James et al., 2020; Swaminathan and Schellenberg, 2020; Lippolis et al., 2022; Jacobs et al., 2024).

Last, but not least, there are several human conditions in which multisensory processing seems to be disrupted, such as autism, schizophrenia, dyslexia, traumatic brain injury, post-traumatic stress disorder, and sensory processing disorder (Hahn et al., 2014; Zvyagintsev et al., 2017; Stein et al., 2019; Wallace et al., 2020; Toumaian et al., 2024). Various research works have also documented significant reductions in auditory MMN amplitude in patients with schizophrenia (Umbricht et al., 2003; Koshiyama et al., 2020), dyslexia (Baldeweg et al., 1999; Gu and Bi, 2020), and autism spectrum disorders (Lassen et al., 2022). How such disorders associate with defects in the integration of sensory information remains to be determined. Nevertheless, it would be interesting to explore whether multisensory learning could yield comparable advantages in the multisensory capabilities of these patients and whether any improvement in their symptoms can be discerned.

### Limitations

One limitation of the present study concerns the use of two different EEG caps during data collection. It is worth noting that the use of the two caps was counterbalanced across participants to minimize any systematic influence on the results. Alongside, the EEG data were analyzed in source space instead of sensor space, and hence the effects of the different caps were explicitly modeled and taken into account prior to Beamformer estimation. To further address this concern, we conducted an additional ANCOVA within the Network-Based Statistics framework to test whether cap type interacted with our main effect of interest (group × time × condition). This analysis revealed no significant influence of cap type, confirming that the reported results are not dependent on the choice of EEG cap. A further limitation arises from the correlational nature of brain connectivity research. As with all neuroimaging studies, it is not possible to disentangle whether neural changes drive behavioral outcomes or whether observed connectivity modifications reflect variations in behavior. Establishing causal relationships between neural activity and behavioral changes would require neuromodulation techniques, which were beyond the scope of the present study.

### Future prospects

It is imperative for future research to explore the neurophysiological pathways of multisensory integration and disentangle the unique characteristics of multisensory training that render it a catalyst for neuroplasticity. Further multivariate analyses could shed light on the exact mechanism of information merging among the different cortical and subcortical regions. At the same time, combining the information acquired simultaneously by electrophysiological and neuroimaging methods, known as multimodal imaging, may incorporate the strong points of each modality, which is the localization ability of imaging solutions (e.g., fMRI) and the temporal resolution of neurophysiological methods (e.g., M/EEG), allowing cortical network modeling with greater precision. These avenues for further study hold promise for advancing our knowledge of the intricacies of the human brain and its wiring mechanisms.

## Conclusion

The present research work aimed to investigate the neuroplastic effects elicited by unisensory and multisensory training on effective connectivity networks that subserve visual, auditory, and audiovisual perception. The outcomes of the study showcased that the cross-modal training altered remarkably the effectivity networks in all three modalities, especially in the audiovisual, whilst the unisensory methodological approach exerted a slight impact solely on the auditory perceptual system. As regards the visual and auditory integrative processes following the multisensory training, the reconfiguration of the connections concerned mainly higher-order cortical areas, suggesting a top-down process of affecting unisensory perception. The regions that exhibited the most frequent alterations are the left MFG, left IFS, and left insula, renowned for their established multisensory attributes, which substantiates the findings indicating their susceptibility to the influence of cross-modal training. Hence, the results of our research not only point to the supremacy of multisensory training compared to unisensory, but also illustrate the top-down mechanism by which the multisensory learning methods affect the integration of senses.

## Data Availability

The datasets presented in this study can be found in online repositories. The names of the repository/repositories and accession number(s) can be found below: https://git@gin.g-node.org/parasvag/MusicPlast.git.

## Footnotes

1
www.neurobs.com

2
http://parasvag.webpages.auth.gr/MusicPlast/game1.php

3
https://www.adobe.com/products/captivate.html

4
http://parasvag.webpages.auth.gr/MusicPlast/game2.php

5
http://neuroimage.usc.edu/brainstorm
